# Multidimensional synergistic adaptation enhances the systemic resilience in China’s food security

**DOI:** 10.1093/nsr/nwaf587

**Published:** 2025-12-26

**Authors:** Yujie Liu, Jiahao Chen, Wenjing Cheng, Xuhui Wang, Tao Pan, Junjie Liu, Yang Lu, Ermei Zhang, Shuyuan Huang, Jie Zhang, Da Lv, Qinghua Tan, Jie Chen, Chenzhi Wang, Yuhao Zeng, Hanchen Wang, Josep Peñuelas, Yong-guan Zhu, Christoph Müller, Jiabao Zhang, Shaozhong Kang, Sien Li, Jikun Huang, Wei Xie, Wenbin Wu, Jonas Jägermeyr, Yan Zhu, Petr Havlik, Jinfeng Chang, Tao Lin, Bing Yu, Shilong Piao

**Affiliations:** Key Laboratory of Land Surface Pattern and Simulation, Institute of Geographic Sciences and Natural Resources Research, Chinese Academy of Sciences, Beijing 100101, China; University of Chinese Academy of Sciences, Beijing 100049, China; Key Laboratory of Land Surface Pattern and Simulation, Institute of Geographic Sciences and Natural Resources Research, Chinese Academy of Sciences, Beijing 100101, China; University of Chinese Academy of Sciences, Beijing 100049, China; Key Laboratory of Land Surface Pattern and Simulation, Institute of Geographic Sciences and Natural Resources Research, Chinese Academy of Sciences, Beijing 100101, China; University of Chinese Academy of Sciences, Beijing 100049, China; Sino-French Institute for Earth System Science, College of Urban and Environmental Sciences, Peking University, Beijing 100871, China; Institute of Carbon Neutrality, Peking University, Beijing 100871, China; Key Laboratory of Land Surface Pattern and Simulation, Institute of Geographic Sciences and Natural Resources Research, Chinese Academy of Sciences, Beijing 100101, China; University of Chinese Academy of Sciences, Beijing 100049, China; Key Laboratory of Land Surface Pattern and Simulation, Institute of Geographic Sciences and Natural Resources Research, Chinese Academy of Sciences, Beijing 100101, China; University of Chinese Academy of Sciences, Beijing 100049, China; Key Laboratory of Land Surface Pattern and Simulation, Institute of Geographic Sciences and Natural Resources Research, Chinese Academy of Sciences, Beijing 100101, China; University of Chinese Academy of Sciences, Beijing 100049, China; Key Laboratory of Land Surface Pattern and Simulation, Institute of Geographic Sciences and Natural Resources Research, Chinese Academy of Sciences, Beijing 100101, China; University of Chinese Academy of Sciences, Beijing 100049, China; Key Laboratory of Land Surface Pattern and Simulation, Institute of Geographic Sciences and Natural Resources Research, Chinese Academy of Sciences, Beijing 100101, China; University of Chinese Academy of Sciences, Beijing 100049, China; Key Laboratory of Land Surface Pattern and Simulation, Institute of Geographic Sciences and Natural Resources Research, Chinese Academy of Sciences, Beijing 100101, China; University of Chinese Academy of Sciences, Beijing 100049, China; Key Laboratory of Land Surface Pattern and Simulation, Institute of Geographic Sciences and Natural Resources Research, Chinese Academy of Sciences, Beijing 100101, China; University of Chinese Academy of Sciences, Beijing 100049, China; Key Laboratory of Land Surface Pattern and Simulation, Institute of Geographic Sciences and Natural Resources Research, Chinese Academy of Sciences, Beijing 100101, China; University of Chinese Academy of Sciences, Beijing 100049, China; College of Marine Ecology and Environment, Shanghai Ocean University, Shanghai 201306, China; Sino-French Institute for Earth System Science, College of Urban and Environmental Sciences, Peking University, Beijing 100871, China; Leibniz Centre for Agricultural Landscape Research, Müncheberg 15374, Germany; Key Laboratory of Land Surface Pattern and Simulation, Institute of Geographic Sciences and Natural Resources Research, Chinese Academy of Sciences, Beijing 100101, China; University of Chinese Academy of Sciences, Beijing 100049, China; Key Laboratory of Land Surface Pattern and Simulation, Institute of Geographic Sciences and Natural Resources Research, Chinese Academy of Sciences, Beijing 100101, China; University of Chinese Academy of Sciences, Beijing 100049, China; Global Ecology Unit CREAF-CSIC-UAB, Center for Ecological Research and Forestry Applications - National Research Council, Edifici C, Universitat Autonoma de Barcelona, Bellaterra 08193, Spain; State Key Laboratory of Urban and Regional Ecology, Research Center for Eco-Environmental Sciences, Chinese Academy of Sciences, Beijing 100085, China; University of Chinese Academy of Sciences, Beijing 100049, China; Key Laboratory of Urban Environment and Health, Institute of Urban Environment, Chinese Academy of Sciences, Xiamen 361021, China; Potsdam Institute for Climate Impact Research, Member of the Leibniz Association, Potsdam 14473, Germany; State Experimental Station of Agro-Ecosystem in Fengqiu, Institute of Soil Science, Chinese Academy of Sciences, Nanjing 210008, China; University of Chinese Academy of Sciences, Beijing 100049, China; Center for Agricultural Water Research in China, China Agricultural University, Beijing 100083, China; Center for Agricultural Water Research in China, China Agricultural University, Beijing 100083, China; China Center for Agricultural Policy, School of Advanced Agricultural Sciences, Peking University, Beijing 100871, China; China Center for Agricultural Policy, School of Advanced Agricultural Sciences, Peking University, Beijing 100871, China; Institute of Agricultural Resources and Regional Planning, Chinese Academy of Agricultural Sciences, Beijing 100081, China; Key Laboratory of Agricultural Remote Sensing, Ministry of Agriculture, Beijing 100081, China; Columbia University Climate School, New York, NY 10025, USA; NASA Goddard Institute for Space Studies, New York, NY 10025, USA; National Engineering and Technology Center for Information Agriculture, Jiangsu Key Laboratory for Information Agriculture, Jiangsu Collaborative Innovation Center for Modern Crop Production, Nanjing Agricultural University, Nanjing 210095, China; Jiangsu Academy of Agricultural Sciences, Nanjing 210014, China; International Institute for Applied Systems Analysis, Laxenburg 2361, Austria; College of Environmental and Resource Sciences, Zhejiang University, Hangzhou 310058, China; College of Biosystems Engineering and Food Science, Zhejiang University, Hangzhou 310058, China; Sino-French Institute for Earth System Science, College of Urban and Environmental Sciences, Peking University, Beijing 100871, China; Sino-French Institute for Earth System Science, College of Urban and Environmental Sciences, Peking University, Beijing 100871, China; Institute of Carbon Neutrality, Peking University, Beijing 100871, China; Key Laboratory of Alpine Ecology, Institute of Tibetan Plateau Research, Chinese Academy of Sciences, Beijing 100101, China; Center for Excellence in Tibetan Earth Science, Chinese Academy of Sciences, Beijing 100101, China

**Keywords:** food security, systemic resilience, multidimensional synergistic adaptation, agricultural advance, climate change

## Abstract

How to manage the compounding risks to national food security is a major issue of global concern. China, as the world’s largest producer of staple foods, has steadily strengthened its food security level, profoundly impacting global food systems. In this review, we propose a systemic resilience framework (the ability to predict, absorb, rebound from and adapt to disruptions) to analyze the evolution of China’s food security and explore its driving factors and multidimensional adaptations. China’s food security resilience has progressed through three distinct stages: low resilience (achieving basic sufficiency), medium resilience (achieving nutritional adequacy) and above-medium resilience (embracing sustainability). Multidimensional synergistic adaptation—integrating agricultural, climatic, socioeconomic and land-use strategies—has been key to these achievements. While agricultural advancements have significantly bolstered China’s food security, the growing pressures of climate change threaten to undermine these achievements. We project that China’s staple food self-sufficiency will remain above 98%, yet the overall food balance is expected to tighten under the combined pressures of dietary shifts and resource constraints. To better enhance the systemic resilience in China’s food security, China can buffer climate- and water-related shocks by expanding high-standard farmland, ease resource and demand pressures by enforcing anti-food-waste laws, strengthen soil and water resilience through nature-based solutions, and dampen trade volatility with integrated climate–market early-warning systems. Insights from China’s experience provide targeted levers for enhancing food-system resilience elsewhere.

## Teaser

China has progressively strengthened its food security—from ensuring basic sufficiency to achieving nutritional adequacy and now pursuing long-term sustainability—through integrated strategies spanning agriculture, climate, society, and land use. This review reveals how these interconnected efforts have built robust systemic resilience and offers practical lessons for safeguarding global food systems against escalating climate and resource pressures.

## INTRODUCTION

Food security is critical to the survival and development of human society, and core to achieving Sustainable Development Goals (SDGs), particularly SDG 2 (Zero Hunger). Although global food production has more than tripled since the 1960s [1], the number of people affected by hunger rose globally to as many as 700 million in 2024. More importantly, over 2.3 billion people across the globe face moderate or severe food insecurity [2]. With rising challenges from a growing population, consumption habits, armed conflicts,

climate change and global pandemics like COVID-19, there is growing concern that the evolution of food security is deviating from the track of achieving zero hunger. Countries around the world have been adopting various adaptation measures to address different food security risks and improve their national food security level.

With the increasing risk factors facing global food security, scientists noticed that the food system is one part of the whole planetary system in which the different components interact with each other and generate an aggregate coupling effect that could not be predicted from even the most detailed knowledge of individual parts in isolation. A prime example is the interaction between climate change adaptations and trade policies. A regional climate adaptation, such as breeding drought-tolerant crops to stabilize local yield, may prove effective in isolation. However, if this leads to reduced agricultural water availability for downstream regions (impacting another production component), and simultaneously a national trade policy increases reliance on food imports (a market component), the systemic outcome could be an unintended increase in vulnerability to international market volatility—a risk that was not apparent when examining any single component alone. This underscores the necessity of a systemic framework to analyze food security and a shift from single-dimensional to multidimensional synergistic adaptation.

In this context, we propose the concept of multidimensional synergistic adaptation, which refers to the integrated implementation of strategies across agricultural, environmental, socioeconomic and policy domains to enhance systemic resilience. Systemic resilience here denotes the capacity of the food system to anticipate, absorb, recover from and adapt to shocks and stresses [3]. This review applies this framework to China, the world’s largest food producer, to analyze its food security trajectory and derive lessons for global practice.

As the world’s largest staple foods (rice, wheat and soybeans) producer with 1.4 billion population, the food security of China has undergone drastic and complex changes. The issue of ‘who will feed China’ has been a global concern since 1994 as its rapid population growth and limited agricultural land would outstrip its ability to feed itself and even overwhelm the world’s grain-producing capacity. But China has basically achieved self-sufficiency in grain production since 2013 [4] instead of threatening the global food supply. According to the World Bank in 2025, China produces 24% of the world’s grain output and feeds 18% of the world’s population, with the availability of only 9% of global arable lands and 6% of global renewable water resources [5]. China has also become a major food-aid provider to sub-Saharan African and Central Asian countries, helping eliminate hunger and stabilize the international food trade [6]. In the past few decades, the systemic resilience of China’s food security system has significantly improved.

However, whether China can sustain the increasing demand has been questioned since the late 2010s due to emerging challenges including a growing affluent population, diet diversification, food wastage and recent events of large crop imports. China imported more than 60% of global soybean and a growing amount of maize, wheat and rice in 2021 [7]. Further gains in productivity could be impacted by water shortage, over-fertilization and climate change. Barriers to international trade, such as trade frictions and armed conflicts, will aggravate those pre-existing challenges, jeopardizing the resilience of China’s food security.

A comprehensive understanding of the resilience changes in China’s food security will provide a reference for other countries committed to achieving food security issues, but there has been a lack of systematic synthesis regarding the latest changes in China’s food security, as well as its drivers and perspectives, which have become an important knowledge gap in China, and the global pursuit of sustainable development.

This review takes China as a typical country case and brings out a systemic resilience framework to address this gap by outlining the temporal resilience changes in China’s food security and discussing the driving factors and multidimensional synergistic adaptations behind it, including agricultural advancements, climate change, socioeconomic shifts and land-use changes. Future projections regarding food supply–demand balance and sustainability are synthesized, along with the connection between China’s food security and other SDGs. The synthesis concludes by proposing essential research priorities for advancing our understanding of China’s food security in the pursuit of sustainable development. Against faltering global progress toward SDG 2, with more than two billion people experiencing moderate or severe food insecurity, we take China as a high-capacity case to investigate pathways for building food-system resilience at scale. Theoretically, we offer an operational resilience framework by linking availability, access, utilization and stability to three stages and drivers for comparing differences across countries. Practically, we provide specific policy implications (e.g. high-standard farmland for heat–drought/water stress, anti-food-waste for demand pressure, nature-based solutions for soil/water resilience, and integrated climate–market early warning for trade volatility) for the rest of the world to make more enlightened policy decisions that promote resilience.

## CHANGING RESILIENCE IN CHINA’S FOOD SECURITY OVER PREVIOUS DECADES

**Figure 1.**
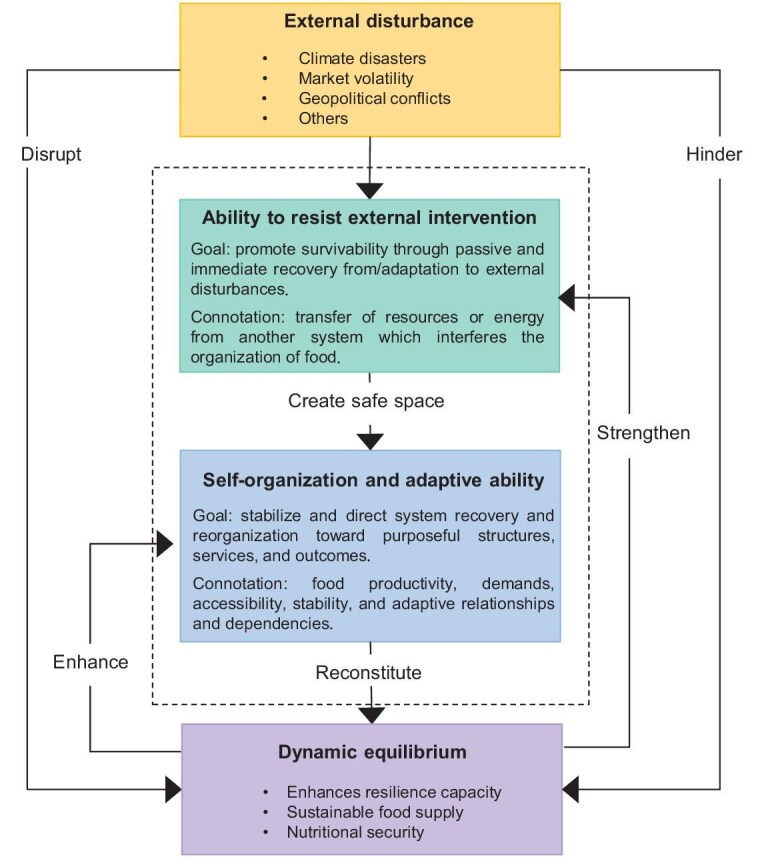
Conceptual framework of systemic resilience in food security. External disturbances (e.g. climate disasters, market volatility, geopolitical conflicts etc.) impose shocks on the food system. The system first relies on its ability to resist external intervention to buffer immediate impacts, and then on its capacity for self-organization and adaptation to stabilize, redistribute resources and restore basic functions. Through this reconstitution process, the system can approach a dynamic equilibrium in which resilience capacity is strengthened and a sustainable food supply and nutritional security are maintained. This equilibrium is not a static end state but a working state that can itself be disrupted by subsequent shocks.

We propose the conceptual answer for addressing food security risks is to strengthen systemic resilience, by which we mean the capacity of the food system to anticipate, absorb, recover from and adapt to a wide array of systemic threats. The framework in Fig. 1 underpins the analysis of China’s three resilience stages, each characterized by distinct external pressures and adaptive capacities. The ‘low resilience’ stage was dominated by responses to external disturbances to create a safe space for survival, while the subsequent stages increasingly relied on self-organization to reconstitute the system towards more sustainable and nutritious steady states. Building on this framework (Fig. 1), we analyze the historical evolution of China’s food security since the mid-20th century, which can be divided into three distinct stages characterized by differing resilience capacities and primary goals: the ‘low resilience’ stage (before ∼2000), which prioritized ensuring an adequate quantity of food; the ‘medium resilience’ stage (∼2000 to ∼2010s), characterized by a transformation in the national dietary structure and food consumption patterns; and the ‘above-medium resilience’ stage (after ∼2010s), which aims to meet the food needs of the present generations without compromising those of future generations (Fig. S1). The rules and indicators for assigning stages are documented in Fig. S1 and the Table S1 [8,9], and mapped to the Food and Agriculture Organization of the United Nations (FAO)’s four-dimension framework in Figs S2–S3. In this section, we further summarize the changes in representative indicators at each stage, including per-capita grain production, self-sufficiency rates, dietary energy intake and composite food security scores.

### Low resilience: from not enough (before 1979) to enough (∼2000)

**Figure 2.**
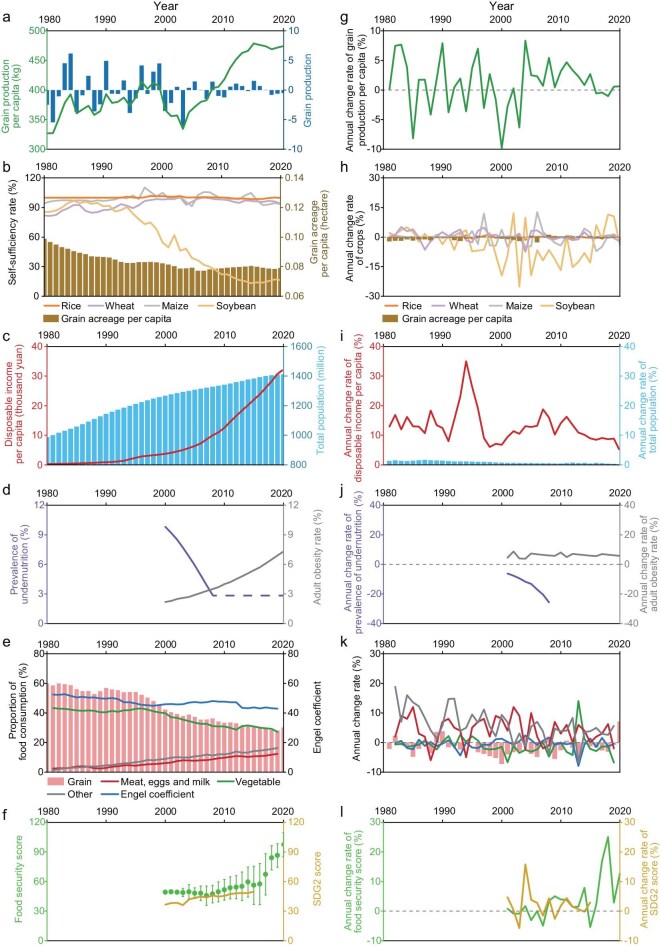
Key indicators of changes of food security in China. To the right side of (a–f), additional figures (g–l) are appended, depicting the annual rate of change for the respective indicators. In (a), the fluctuation of grain production is measured by the change relative to the average of the 5 years centred around the current year. In (b), the self-sufficiency rate is measured by the ratio of production to production plus net imports. In (d), the dashed section indicates an undernutrition rate of less than 2.5% but with strong simulation uncertainty, and obesity means that body mass index is greater than 25, as defined by the World Health Organization.

The primary concern regarding China’s food security at this stage was ensuring a balance in basic grain supply and demand while addressing malnutrition. Here, ‘not enough’ refers to per-capita daily calorie availability below 2100 kcal (vs. the global recommendation of 2500 kcal/day), insufficient per-capita domestic grain supply, and pronounced production volatility; ‘enough’ refers to meeting or exceeding the 2500 kcal threshold (reached by 1993), sustained per-capita grain output of ≥400 kg since 2010, a prevalence of undernourishment (PoU) below 2.5%, and stable self-sufficiency for staple grains. Before 1979, China’s food supply was primarily self-reliant, with per-capita daily calorie availability remaining below 2100 kcal [10], significantly below the global recommended amount of 2500 kcal/day [11]. The low levels of agricultural productivity failed to meet the rising food demands of the rapidly growing population, which increased from approximately 0.5 billion in 1949 to around 1.0 billion in 1979 [12]. In 1979, the implementation of the ‘reform and opening up’ policy, including the household responsibility system, stimulated agricultural productivity [13]. Over the next two decades, China’s grain production steadily increased from 327 kg per capita in 1979 to 406 kg per capita in 2000 due to technological advancements, institutional innovations and increased input of land resources (Fig. 2a and b), aiming to ensure sufficient food for the continuously growing population (Fig. 2c). During this period, food security significantly improved, with a 20% increase in per-capita calorie availability, reaching the global recommended amount by 1993 [13]. However, the per-capita domestic grain supply remained relatively insufficient, and grain production fluctuated intensely (Fig. 2g).

After 2000, fluctuations in grain production decreased, and stability was observed. Since 2010, China’s annual per capita grain production has consistently remained above 400 kg, ensuring ample grain supply to prevent systemic food crises and surpassing the global average by 50% in per-capita grain production, showcasing China’s abundant grain self-sufficiency capabilities [14].

On the demand side, China’s booming economy since the 1990s to the early 21st century led to a doubling of the ratio of disposable personal income (DPI) to the Consumer Price Index (CPI) based on the 1980 benchmark. Consequently, the demand for food steadily increased (Fig. 2i). The Engel coefficient exhibited a significant decline from 57% in 1990 to 30% in 2022 (Fig. 2e), and per-capita daily calorie availability reached 3336 kcal/day in 2020. The growth in income enhanced economic access to food, while extensive transportation and infrastructure constructions increased physical accessibility.

Overall, Chinese people have increasingly been able to obtain sufficient food, reducing the PoU to below 2.5% (Fig. 2d). According to the 2000 Central Economic Work Conference in China, as documented in subsequent government white papers on food security, China had essentially solved the challenge of ensuring adequate food for its rural poor population by the year 2000 [15]. The self-sufficiency rate of staple food (in China, referring to rice and wheat [16]) has remained stable and high (Fig. 2b). However, as demands increased, the domestic supply-and-demand gap for non-staple food such as soybeans has not been filled, leading to continuous increases in imports to meet growing demand (Fig. 2h). This stage was primarily driven by policy reforms (e.g. the household responsibility system), input intensification (e.g. water and fertilizer inputs) and technological advancements (e.g. variety replacement) to achieve the fundamental goal of ‘eating enough’.

### Medium resilience: from enough to eating well (∼2000 to ∼2010s)

Since 2000, with improved agricultural productivity and economic development, China no longer faces food shortages. Consequently, the dietary habits of the Chinese population have evolved, marked by increased consumption of high-protein foods, dairy products and other non-starchy food products (Fig. 2k). The proportion of meat, eggs and milk consumption reached 13% of the total food consumption by weight in 2021, double that of 2000 [17]. For context, this proportion was below 7% before 2000, highlighting the significant dietary shift during this stage. In 2021, per-capita fruit consumption reached 61 kg/year, almost double that of 2000, moving closer to recommended dietary patterns [18]. Per-capita grain consumption decreased by 23%, from 189 kg/year in 2000 to 145 kg/year in 2021 [19].

This diversified dietary structure has led to improvements in nutritional status and health. For instance, the prevalence of low birth weight among infants has gradually declined and remained low. The prevalence of anemia among women has also decreased by over 5% in the 21st century [18]. However, this shift in dietary patterns has introduced new problems, such as obesity. Since the 2000s, the issue of overweight and obesity in China has become increasingly prominent, with the rate rising from below 4% in the 2000s to 16% in 2019 [20] (Fig. 2j). This is because diets became more Westernized (greater intake of animal-source and ultra-processed foods, more eating out) and physical activity declined with longer sedentary time over this period.

### Above-medium resilience: towards eating sustainably (after ∼2010s)

Starting from the 21st century, especially in the 2010s, China has increasingly focused on sustainable agricultural development and the sustainability of food security, owing to the enriched connotation of food security and the deepening ideas of sustainable development [21]. During this period, China’s attention shifted from supply-related aspects to sustainability factors, encompassing sustainable resource utilization and environmental protection.

Integrated assessments of China’s food security sustainability have shown a steady increase in the SDG 2 score and the magnitude of its improvement in the 21st century, involving resource and environmental indicators such as water and land use (Fig. 2f). The Global Food Security Index of China’s natural resources and resilience dimension also demonstrated a 12% improvement in 2020 compared to 2012 [22], indicating progress in the protection and sustainable utilization of natural resources in China’s agriculture sector. The composite score of China’s food security displayed significant increases after 2015 (Fig. 2l, Methods and Table S1), pointing towards a more sustainable trend in food security development.

However, improved food security has also brought new challenges to sustainability, including the diversification of demands and the stress on natural resources and the environment. For instance, to maintain high agricultural production efficiency, the consumption of agricultural fertilizers exceeded 50 million tons in 2022. The fertilizer application per unit area for agriculture remained at 429 kg/ha, significantly higher than the global average (120 kg/ha) [23]. Pesticide application amounted to 8.7 kg/ha in 2019, which was 3.3 times the global average [24]. Intensified agricultural production and excessive use of agrochemicals pose risks to ecosystem services and soil quality. It is important to note that while these sustainability concerns became a central focus during this stage, the intensive application of fertilizers and pesticides was most pronounced from the late 1990s to the 2010s, resulting in the environmental legacy that the ‘above-medium resilience’ stage now seeks to address. Additionally, food waste remains a significant concern, with China generating 56.75 million tons of food waste in 2018 [25].

In response to these pressing sustainability concerns, various strategic management measures will be implemented. Tailored nitrogen management strategies, for instance, can enhance farm profitability, reduce field ammonia emissions and improve air quality simultaneously.

## KEY DRIVERS OF MULTIDIMENSIONAL SYNERGISTIC ADAPTATIONS TO CHINA’S FOOD SECURITY RESILIENCE CHANGES

**Figure 3.**
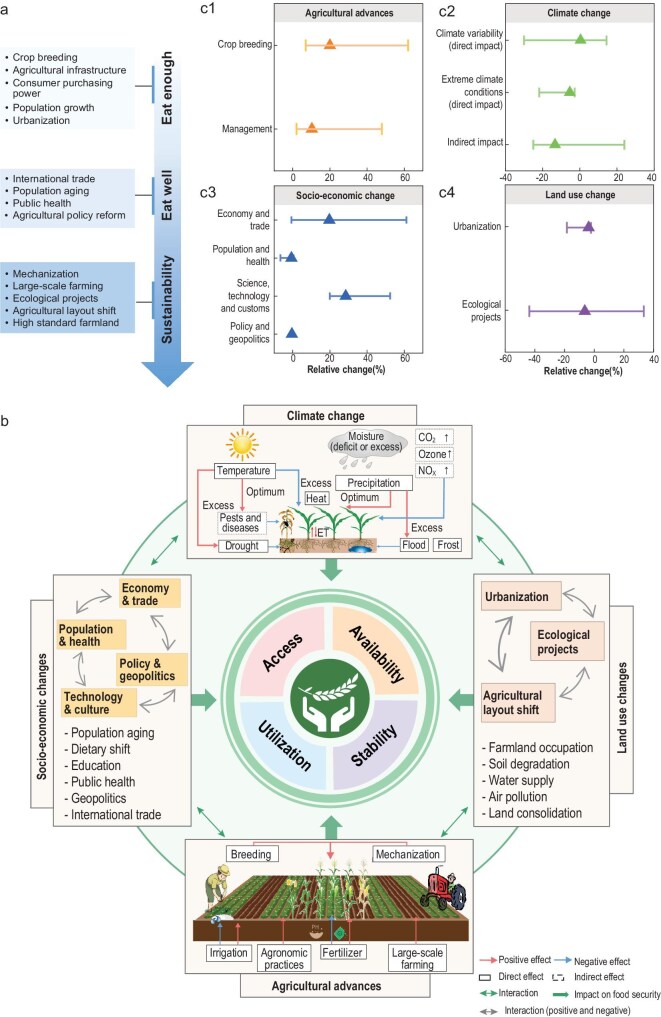
Key drivers and adaptations in China’s food security resilience changes. (a) The blue arrows represent the three stages of China’s food security: ‘low resilience’, ‘medium resilience’ and ‘above-medium resilience’. (b) The four boxes represent agricultural management, climate change, socioeconomics and land use, respectively, and the middle circle represents the four pillars of food security. Arrow colors indicate which pillar is affected by each driver. Within the climate-change panel, red lines indicate a positive or strengthening effect, whereas blue lines indicate a negative or constraining effect. For example, increasing temperature can intensify drought occurrence (a positive effect on drought), while drought in turn suppresses crop growth and development (a negative effect on yield and resilience). The color of the arrows pointing to the circle represents the dimension affected by the driving factor. (c) The quantitative impact of driving factors on the yield. Triangles represent the median, and the left and right horizontal lines represent the maximum and minimum values. Plot data for c1, c2, c3, c4 are derived from the literature.

Changes in China’s food security have been influenced by four interconnected categories of drivers and adaptations: (i) agricultural advances; (ii) climate change; (iii) socioeconomic development; and (iv) land use changes (Table 1 and Fig. 3). Among these, there are both natural and human drivers, as well as adaptive measures taken by humans to reduce adverse impacts. Sometimes, it is difficult to simply distinguish the synergistic effects of different drivers and adaptation measures on food security production. In this section, we synthesize the contribution of key drivers and adaptations together using meta-analysis (Supplementary Methods, Fig. S4a) to quantitatively attribute past changes in food security to different factors based on existing literature.

**Table 1.** Drivers and multidimensional synergistic adaptations to China’s food security.

Drivers and adaptations	Description
Agricultural advances	Crop variety breeding and agricultural management including fertilization, irrigation, mechanization and large-scale farming etc.
Climate change	Cultivar shifts, optimizing sowing windows, and optimized planting density etc.
Socioeconomic development	GDP growth, population increase, advancements in education, science and technology, and changes in customs etc.
Land-use change	Rapid urbanization, national ecological restoration projects

### Agricultural advances

Crop yield has been significantly increased, which is largely driven by agricultural advances, involving crop variety breeding and agricultural management (fertilization, irrigation, mechanization and large-scale farming). Variety breeding and agricultural management contributed 7%–62% and 2%–48% to yield improvement, respectively (Fig. 3a). The contribution of breeding has varied over time but stabilized recently.

Variety breeding has led to substantial improvements in stress tolerance, photosynthetic efficiency and harvest index, and thus the crop yield. The average contribution of crop variety breeding to increased yield in China by 2020 was estimated to be about 45% [26], varying across crops and with time. Notably, the contribution of variety breeding to yield appeared to decline during 1950–2000, as indicated by growingly smaller genetic yield gains of summer maize cultivated in the North China Plain (from 44% to 21%), but has been stabilized through continuous innovation in recent decades. Hybrid technologies and genomic design have been integrated into the breeding of high-yielding crop varieties that also feature broad ranges of tolerance to environmental conditions, pathogens and insect pests. By the end of 2020, the coverage rate of improved crop varieties had exceeded 96% [27]. For example, in southern China, the widespread use of hybrid rice resulted in an approximately 19% higher yield than inbred rice [28]. There have been four to five cycles of maize hybrid replacement in China since the 1970s, with 53% of the yield increase attributed to breeding breakthroughs [29]. Breeding of super hybrid rice [30] has increased yield potential by 12% compared with ordinary hybrids and inbred varieties. Notably, the contribution of variety breeding to yield appeared to decline during 1950–2000, as indicated by growingly smaller genetic yield gains of summer maize cultivated in the North China Plain (from 44% to 21%) [31].

Progress in agricultural management, including optimization of fertilizer use and irrigation, mechanization and large-scale farming, have greatly improved the efficiency of agricultural production. Improvements in agricultural management led to a divergent rate of rice yield increments (13%) and wheat yield increments (6%) from 1990 to 2019 [32]. Higher yield increment for rice can be attributed to its greater responsiveness to improved water and nutrient management under intensive cropping systems. China has experienced a 3-fold increase in synthetic nitrogen (N) fertilizer application from 1982 to 2017 [33]. This surge in N fertilizer application has boosted crop production in China and contributed around 45% ± 3% of increases in crop yields from 1955 to 2014 [34]. Irrigated croplands have accounted for 50% of China’s total croplands and produced approximately 75% of food and over 90% of industrial crops [35]. Improvement in agricultural water use and conservancy have effectively mitigated negative effects of climate change and sustained crop production, especially in water-limited regions. As an example, drip irrigation has demonstrated a reduction of 23% in irrigation water usage and a 7% improvement in water use efficiency in Northwest China [36].

Larger farm sizes, growing mechanization and the ongoing transformation from traditional agriculture to smart agriculture increased crop production nationwide, through enhanced agricultural resource optimization and elements allocation [23]. Enlarging farm size has enhanced agricultural productivity, as well as contributing to the sustainability of agricultural production. For smallholder agriculture, which is widely dispersed in southern China, the establishment of large-scale farming systems has reduced labour requirements by 39%, while doubling labour incomes [37]. High standards of farmland can greatly improve resource-use efficiency and increase food production. Thirty percent of reclaimed land parcels increased productivity [38]. From 1979 to 2020, China’s integrated mechanization rate of crop planting and harvesting has increased to 71%, with the mechanization rates of wheat, rice and maize at 97%, 84% and 90%, respectively [39]. For example, modernizing smallholder farms in Jiangsu Province demonstrated an average yield increase of approximately 8%, with observed gains ranging from 2% on less suitable land to 13% on optimally managed farms, and reduced total nitrogen fertilization use by 3%–13% [40]. Leveraging remote sensing, big data, artificial intelligence and other cutting-edge technologies, there is increasing application of smart and precision agriculture, which promotes food production by increasing the adaptive capacity of farmers, as well as increasing resilience and resource use efficiency.

### Climate change impacts and adaptations

Placing China’s climate impacts in a global context, they have been found to affect China’s food availability and stability through multiple direct and indirect pathways. Direct impacts, including changes in mean climate and extreme climate events, accounted for −30% to 14% and −22% to −3% of yield variation, respectively, while indirect effects (including changes in atmospheric components, crop pests and diseases) accounted for −25% to 24% of crop yield changes (Fig. 3b). These impacts and adaptation responses are part of a broader global pattern of climate–agriculture interactions.

Rising mean temperature affects crop yields by shortening the growing season and reducing productivity when it surpasses the optimal temperature range. Conversely, it can increase yields in regions where ambient temperatures are initially below the optimum, for instance by extending the growing season in cooler regions such as Northeast China [41]. A synthesis of studies indicates that a 1°C increase in global mean temperature has, on average, led to wheat, rice and maize yield reductions in China by 2.6%, 4.3% and 4.0%, respectively [42]. Nevertheless, rising temperatures have expanded the cultivation area in northern China and increased national production of major grains (wheat, rice and maize) by 2.2% [43] during 1981–2010. Besides warming, changes in precipitation directly impact crop yield by altering moisture stress and irrigation water demands and availability [44]. A 100 mm increase in total precipitation during the growing season resulted in a 4% increase in maize yield, accounting for a 20% yield increase for maize during 1982–2013 [45]. Conversely, extreme precipitation can lead to waterlogging damage, particularly in poorly drained soils [46].

The increasing frequency, intensity and extent of climate extremes are growing concerns for food security. Nationwide observations provide evidence of increasing impacts of extreme climate events on yield reduction in China. Extreme heat, drought, cold and other events (such as hail, typhoons and tropical cyclones) respectively resulted in rice yield reductions of 5.4%, 4.2%, 3.7% and 2.9% during 1999–2012 [46]. Exposure to one extra day of extreme heat (above 33°C, with some regions experiencing temperatures far exceeding this threshold during heatwaves) decreased China’s agricultural total factor productivity (TFP) by nearly 2% [47], reflecting the ratio of measured output (such as crops, livestock, and goods and services) per unit of measured inputs (such as land, labor, capital and resources). The major food basket in Northeast China suffered more from droughts than the national average, with moderate and severe drought events during 1961–2017 accounting for 3% and 22% yield losses in maize, and 10% and 14% yield losses in soybean, respectively [48]. Notably, extreme precipitation is often dismissed but is a critical factor. Previous research has demonstrated that extreme precipitation in China between 1981 and 2012 accounted for a 7.6% decrease in rice yield [46], equivalent to those induced by extreme heat.

Changes in atmospheric components, crop diseases and pests caused by climate change impact food security indirectly. Elevated carbon dioxide (CO_2_) concentrations enhance plant water-use efficiency and carbon fixation to mitigate the adverse effects of climate change. Evidence from experimental and modeling analysis showed that an increase in CO_2_ up to 550 parts per million (ppm) enhanced C3 crop (rice, wheat and soybeans) yields by an average of 24% relative to ambient CO_2_ [49]. China’s CO_2_ has increased by 65 ppm since the 1990s [50]. However, the contribution of increased CO_2_ to crop yields in China remains unclear, as CO_2_ is markedly uniform across the globe and there is no consistent spatial variation on which to estimate yield responses to increasing CO_2_ [49].

High levels of ozone entering crop leaves through stomata induce oxidative stress and negatively affect crop development. Ozone levels were estimated to suppress wheat yields by as much as 25% averaged over 2010–12 in China [51]. Similarly, nitrogen dioxide (NO_2_) affects crop yields by directly damaging plant cells and indirectly promoting ozone and aerosol formation. A negative correlation between NO_2_ and crop growth was detected during 2018–20. Reducing nitrogen dioxide emissions to the current fifth-percentile levels increased winter crop yields in China by roughly 25% [52], although this assessment used national-scale statistics. Climate change accounted for 22% of the observed increase in the occurrence of crop pests and diseases during 1970–2016 [53]. Annual losses of crop yield due to pests and diseases increased from about 6 million tonnes in the early 1970s to about 13 million tonnes in the mid-2000s [44].

Adaptation measures to climate change (for example, cultivar shifts, optimizing sowing windows [54]) have so far been effective in mitigating the negative effects of climate change. Historically, the potential benefits of adaptations to wheat yield in temperate and tropical systems were about 18% [55]. The positive impact of climate change on maize yields (15%–30%) [56] in the North China Plain was higher when adaptation measures were considered (8%) [57]. Earlier sowing dates resulted in a maize yield increase of up to 4%, and the adoption of longer growth duration cultivars led to a substantial boost in yield, ranging from 13% to 38% in Northeast China during 1981–2007 [58]. Moreover, optimized planting density in combination with reduced nitrogen application rates has increased maize yield by 6.6% [59]. In Northeast China, the promotion of conservation tillage, while requiring more than 10 different models to suit local conditions, has been identified as a key adaptation strategy to improve soil health and climate resilience [48].

### Socioeconomic development

The rapid socioeconomic development in China, including gross domestic product (GDP) growth, population increase, advancements in education, science and technology, and changes in customs, has driven steady improvements in food security, particularly since 2000 (Fig. 3). Previous studies estimate that socioeconomic changes contributed to food security from −7% to 61% (Fig. 3c). The negative contribution in some contexts may reflect the resource and environmental costs associated with rapid economic growth.

China’s average annual GDP growth rate was 9% from 1990 to 2024 in mainland China, with agricultural GDP increasing by 4% annually [60]. This led to the efficient operation of food markets and easier access to foods. Rising incomes increased consumer purchasing power, fostering a demand for dietary diversity and nutritional quality [52], resulting in changes in agricultural production structures. Moreover, increased agricultural inputs and investment in agricultural infrastructure improved food availability and facilitated domestic food trade, enhancing food stability. For instance, between 2000 and 2017, irrigation, electricity and road infrastructure increased by 33.8, 4.5 and 2.4 times, respectively [61], effectively enhancing resilience against climate change. Growing domestic demand also boosted China’s crop imports, with soybean import dependence averaging 87% during 2017–24 [62]. International trade increasingly affects China’s food security through production, prices and supply chains, and these impacts expand as China’s economy grows [6].

Population growth also led to increased food demands, posing a growing challenge to food security. China’s population growth rate exceeded 8.2‰ in the 1990s [63] and ranged between 4.8‰ and 7.6‰ in the 2000s. Gradual declines in population growth (6.1‰ in 2011 and −0.99‰ in 2024) helped reduce pressure on food demand after the 2010s [63]. Population demographics and public health in China have influenced the sustainability of food production. For example, an aging rural population reduced labor productivity and increased land desolation, resulting in a 0.9‰ decrease in food consumption and a 0.4% reduction in food production with each 1% increase in the aged population [64].

Education, science and technology, and customs positively affect food security. Over the past decade, China’s education level continued to rise, and the illiteracy rate dropped from 4% in 2010 to 3% in 2020 [65]. It is estimated that improved access to primary education reduced food insecurity by around 20%–25% [66], promoting the application of new technologies in agriculture and a transition in dietary preferences to access more nutrient-dense foods. Advances in agricultural science and technology, such as smart irrigation systems and improved food processing, together with broader science and technology—cold-chain logistics, rapid food-safety sensing and traceability, shelf-life-extending packaging and digital distribution platforms—have contributed to building a society where people eat well by enhancing productivity, availability, food safety and nutritional qualities. Thrift and anti-waste customs have been core principles in China’s eating habits, promoting sustainable eating practices. However, food waste remains a significant issue in China, with surveys indicating that food loss and waste reach almost 30% [67].

To encourage food production and ensure self-sufficiency since 1990, China has implemented a series of agricultural policy reforms, including abolishing agricultural taxes, increasing agricultural subsidies and introducing rural social service programs, which have boosted agricultural production [21]. To ensure food security sustainability with limited resources, the Chinese government deepened its agricultural subsidy system reform with a green and ecological orientation, resulting in a 9% growth in China’s TFP [68]. TFP growth, which captures output per unit of total inputs (land, labor, capital and materials), has become an increasingly important source of agricultural growth in China, reflecting improved efficiency. Changing international situations, such as trade frictions between China and the USA and the conflict between Russia and Ukraine, are driving price inflation for imported food, energy, seeds and fertilizer, posing rising risks to global and national food supplies. China has also formulated relevant development plans for international cooperation in agriculture, optimizing the structure of foreign investment in agriculture and promoting food-aid projects, actively responding to current negative impacts from increasing trade barriers. Recent research highlights China’s proactive adaptation to geopolitical conflicts through diversified international trade partnerships and strategic grain reserves [69]. For instance, China has increased agricultural cooperation with Belt and Road Initiative countries, reducing reliance on single-source imports. Meanwhile, in 2020 China established the Lancang–Mekong Agricultural Cooperation Guangxi Sub-center and has since implemented 60+ projects across Vietnam, Laos, Cambodia and Myanmar, including China–ASEAN crop-variety trial stations that report 20%–50% yield gains.

### Land-use change

Land-use change, including rapid urbanization and national ecological restoration projects, has contributed between −44% and 33% to food security (Fig. 3d).

Urbanization has often been viewed as a threat to food security, due to the encroachment of cropland. Since 1980, nearly 3.0 million ha of cropland in China has been converted to urban use and represents 50% of overall national losses in cropland (5.9 million ha) [70]. The loss of arable land—particularly the conversion of high-quality, productive cropland to urban uses—has generated marginal and fragile farmland, often of lower quality and productivity, typically located on slopes or with poor soil conditions, and has led to a subsequent 19% drop in farmland per capita [71]. In addition to land conversion, waste disposal and urban air pollution caused by soil pollution have rendered 2.5% of arable land (3.3 million ha) uncultivable, reducing the productive potential and sustainability of China’s agroecosystems [71]. The rural population decline caused by urbanization cannot be ignored. Higher urban employment wages have enticed a large number of rural youths to leave the countryside, which has led to the reduction of the agricultural labor force and the abandonment of arable land. To adapt to land-use change driven by rapid urbanization, China has adopted a sequenced portfolio. It first safeguards quantity and prime soils through the 1.8-billion-mu cropland red line and permanent basic farmland; it then maintains a stable quantity–quality baseline via occupation–compensation balance and large-scale high-standard farmland construction; it curbs conversion pressures by rectifying ‘non-agricultural’/‘non-grain’ uses and enforcing urban growth boundaries; and it optimizes spatial allocation and resilience through region-specific planting in the nine agro-regions and a ‘big-food’ framework that diversifies supply. Together, these measures buffer food security against ongoing land shifts.

National ecological restoration projects (ERPs) such as the Grain to Green Program, have unintended and considerable impacts on crop production. Similar to urbanization, ERPs could also reduce cropland area. About 0.80 million ha arable land has been converted to forest (0.30 million ha) and grassland (0.50 million ha) in China’s drylands since 2000 owing to several dryland conservation and restoration programmes [72]. Although ERPs reduce the total area of croplands, productivity of the transformed cropland tends to be low, as they are located on sandy soils or steep slopes. Quantitative assessments indicate that ERP-induced cropland conversion has directly reduced grain production by approximately 2%–5% in affected regions, partially compensated by higher yield on remaining land. However, improved soil quality and microclimates in land included in ERPs can increase crop yield [73]. It should be noted that ERPs not only affect local croplands, but also affect the downstream croplands of the river basin by altering run-off. The increasing vegetation cover over bare or sparsely vegetated area could consume more soil moisture, resulting in a decrease in available water supply for regional crop production.

The drivers and adaptations mentioned above do not operate in isolation but interact in complex ways, thus producing a mixture of synergies and trade-offs. For example, agricultural advances, such as drip irrigation, work synergistically with land-use policies that safeguard water resources and with climate adaptation strategies that optimize water utilization. Conversely, the expansion of bioenergy crops may compete with food crop area, highlighting the need for integrated land-use planning. Understanding these interactions is central to designing effective multidimensional synergistic adaptation strategies.

## FUTURE FOOD SECURITY IN CHINA UNDER MULTIDIMENSIONAL ADAPTATIONS

Projecting the evolution of food security in China poses a significant challenge due to the uncertainties surrounding future driving forces, such as policy designs and geopolitical changes, coupled with the complexity of driving mechanisms. Building on the findings in the previous section, we examine how documented climate effects, together with socioeconomic trends, shape China’s future food supply–demand balance.

### Projected changes in the balance between food supply and demand

**Figure 4.**
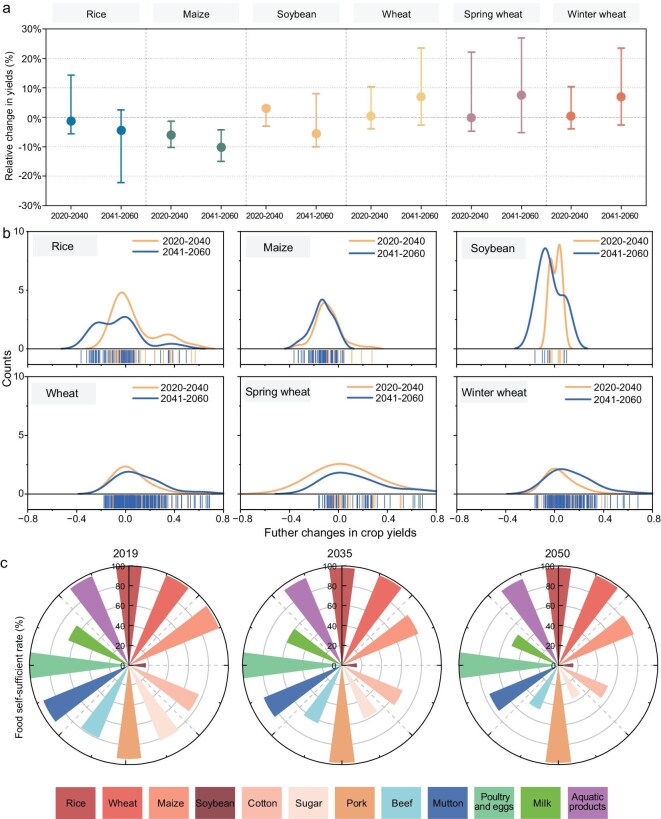
Future food security in China. (a) Future changes in crop (rice, maize, soybean, wheat, spring wheat, winter wheat) yields in China. I-box plot dots indicate median, upper and lower boundaries are 25% and 75% quartiles. (b) Probability distribution of future changes in crop (rice, maize, soybean, wheat, spring wheat, winter wheat) yields in China. (c) Projections of China’s food self-sufficiency based on the CAPSiM model.

The future food supply in China will be influenced by climate change, socioeconomic change, land-use change, agricultural advances and other factors. Our meta-analysis integrating findings from multiple modeling studies demonstrates that future climate and socioeconomic changes will create divergent trajectories for four major crops (Fig. 4a and b). Without additional adaptation beyond current practices, rice yields will decline by 1.2% and 4.3% in the 2030s and 2050s, respectively. Maize yields are projected to decline by 6.0% and 10.1% in the 2030s and 2050s, respectively. Wheat maintains a unique position with projected increases of 0.4% and 7.0% over the same periods, reflecting its physiological advantages under moderate warming.

These crop-specific patterns align with established physiological principles but are moderated by three key modeling uncertainties: (i) the representation of CO_2_ fertilization effects, noting that C4 crops like maize are more susceptible to heat stress during critical reproductive stages, leading to projected yield declines [74]; (ii) parameterizations of crop responses to concurrent heat and water stress; and (iii) the integration of adaptation feedback and socioeconomic drivers [74]. The combined effect of these uncertainties produces an estimated ±10%–15% range around yield projections.

On the production side, China’s agricultural system demonstrates adaptive capacity through continuous productivity gains and food source diversification. Official projections indicate grain output reaching 767 million tonnes by 2032, though this trend faces counterpressures from arable land degradation and external disruptions such as pandemics and armed conflicts [69,75].

Demand-side analysis reveals a structural transformation that will fundamentally reshape China’s food requirements. While population stabilization reduces direct grain consumption, the dietary transition toward animal-based products drives a dramatic increase in feed demand. The demand for oilseed crops is projected to triple from 2010 levels, reaching approximately 200 million tonnes by 2050 [6], creating a fundamentally different demand composition.

Synthesizing these trajectories reveals a tightening balance with distinct implications across food categories (Fig. 4c). We complemented our literature-based evidence with projections from the China Agricultural Policy Analysis and Forecasting Model (CAPSiM), a multi-commodity economic model of China’s agriculture. Under the baseline (status-quo policy) scenario, CAPSiM projects that the national grain self-sufficiency rate will decline from 95% in 2019 to 90% by 2035 [76]. This aggregate trend, however, masks a critical divergence: while staple grains maintain high self-sufficiency (>98%) [77], feed grains show widening deficits, with maize self-sufficiency projected to decline by 16% and soybean dependence remaining near 85% by 2050. This structural imbalance between staple grain security and feed grain dependency represents a core challenge for future food-system resilience.

### Future resilience in China’s food security

Despite entering the ‘above-medium resilience’ phase, China’s food security continues to face persistent challenges that threaten its long-term sustainability. These challenges manifest across three interconnected domains: (i) resource-environment constraints including water scarcity [78], soil degradation projected to cause a 9% productivity loss by 2030 [79] and pollution pressures [37]; (ii) structural imbalances, primarily driven by the growing feed grain deficit; and (iii) systemic vulnerabilities, arising from market volatility and geopolitical tensions. Looking forward, the effectiveness of technological adaptations will be crucial for enhancing resilience. Smart agriculture and climate-resilient crop varieties are projected to mitigate yield losses by up to 15% under moderate climate-change scenarios. Precision agriculture technologies, for instance, could increase water-use efficiency by 20% and reduce fertilizer use by 15% by 2030. However, the successful implementation of these measures depends on timely adoption by farmers, supportive institutional frameworks and continuous investment in research and development.

Policy responses are evolving to address these multidimensional challenges. The development of high-standard farmland is designed to boost production capacity by 10%–20% while enhancing resource efficiency [80], and the Anti-Food Waste Law addresses significant supply chain losses [67]. Technological adaptations show particular promise, with smart agriculture and improved crop varieties potentially mitigating up to 15% of climate-induced yield losses [81], and precision agriculture technologies projected to increase water use efficiency by 20% by 2030 [82].

### Uncertainties in projections

The projections carry unavoidable uncertainties, including underrepresented climate extremes, model-dependent CO_2_ fertilization effects constrained by water and nutrients, structural differences across crop models, and uncertain socioeconomic pathways [83]. Policymakers should interpret the results as scenario-dependent ranges and prioritize robust, adaptive strategies (such as ensemble stress-tests, flexible reserves, and diversified trade) while supporting monitoring and model improvement.

Using our compiled corpus (*n* = 828 crop–scenario samples), we report for each crop and time slice the median, interquartile range and 5%–95% range of projected yield changes. We then compare these yield ranges with population- and consumption-driven demand paths to assess whether staple production can meet future needs. These results provide a transparent fluctuation range for the projections and a simple credibility signal, while recognizing that full probabilistic forecasts would require integrated climate–crop–economic ensembles beyond the scope of this synthesis for policymakers.

## SYNERGISTIC EFFECTS OF CHINA’S FOOD SECURITY TO MULTIPLE SDGS

**Figure 5.**
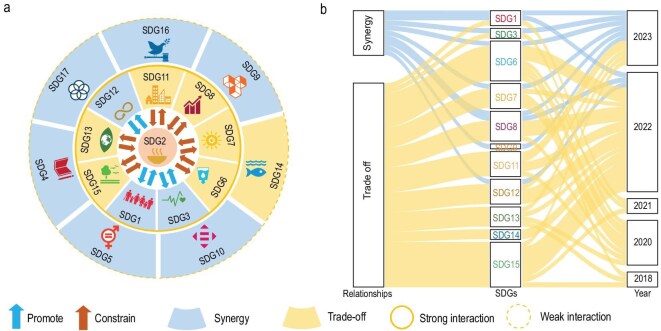
Ensuring food security is the main way to achieve SDG 2 (zero hunger), which has multiple synergies and trade-offs with the other SDGs (the meta-analysis of the data published in 21 peer-reviewed studies, see Fig. S4c). (a) Potential interactions between SDG 2 and other SDGs in China. (b) Interactions between SDG 2 and other SDGs changed dynamically over time in China. SDGs with a solid orange circle outside have a strong interaction with SDG 2. SDGs inside the dashed orange circle have a weak interaction with SDG 2. Blue arrows indicate positive association, whereas red arrows indicate negative or competing effects. The light blue background indicates synergistic effects, and the light orange background indicates trade-off effects.

Achieving zero hunger and improving nutrition (SDG 2) is a prominent goal in the 2030 Agenda for Sustainable Development, which relies on enhancing food security. In this regard, China has achieved significant success, demonstrated by a 36% increase in the national SDG 2 score from 2000 to 2015 [84]. The meta-analysis based on 21 peer-reviewed studies (Fig. S4c) indicated that promoting SDG 2 was strongly connected with 9 of the 16 SDGs, and these relationships may also change over time (Fig. 5).

Realizing SDG 2 and food security has multiple synergies with nine other SDGs (Fig. 5a). For example, universal access to a sufficient and affordable food supply is a prerequisite for poverty eradication (SDG 1), promotion of good health and well-being (SDG 3), quality education (SDG 4) and gender equality (SDG 5) [85]. Bilaterally, remarkable progress in promoting quality education (SDG 4), gender equality (SDG 5) and reducing inequalities (SDG 10) in China has synchronized with improved food security [86]. The education of mothers is crucial for their own nutrition and health, as well as that of their children [87]. In rural China, greater access to education for pregnant women is conducive to reducing the incidence of malnutrition and low weight in children and improving children’s growth [87]. From the perspective of a broader community of shared future for mankind, enhancements in global partnerships (SDG 17) could help exploit the mutual strengths between countries and promote global food security [88]. As a core participant, supporter and contributor of the FAO and the World Food Program (WFP), China has played an active role in efforts to secure global food security, such as providing emergency assistance to regions in food-security vulnerabilities [89].

Connections among SDGs are not immutable but dynamic over time (Fig. 5b). From 2015 to 2018, decoupling processes from synergy to no connections were observed between SDG 2 and some other SDGs, such as SDG 1 and SDG 3 [90]. Across the country, SDG 2 has generally declined, mostly due to food security issues arising from unreasonable consumption patterns, even though food production continued to increase. The synchronism between the decline in SDG 2 and the positive development in poverty reduction (SDG 1) explains the synergistic decoupling.

Advancements toward food security in China could be in trade-off with several SDGs. For instance, reconciling the ever-increasing irrigation demand for increased productivity has become a major challenge for the sustainable management of water (SDG 6) [91]. Widely used neonicotinoid insecticides since 2010, which are prone to be water-soluble in agricultural activities, induced acute or chronic toxicity to aquatic life (SDG 14) along the east coast of China due to discharge into water through run-off and drainage, adversely affecting aquatic ecosystems (SDG 14) [92]. Progress in certain goals, such as clean energy (SDG 7) and economic growth (SDG 8), and mitigation in climate actions (SDG 13), may have a potential trade-off with food security [93]. Globally, bioenergy crop production might be deployed as a solution to the supply of clean energy in the second half of this century, which may reduce the area of cropland for food production, putting as many as 160 million people at risk of hunger in 2050 [94]. Such perspectives on trade-offs have spurred China’s policymakers to limit the conversion of the cultivation of food crops to bioenergy crops [95]. Emerging evidence has indeed deepened concerns about the competition for land due to land-based mitigation strategies like afforestation and bioenergy with carbon capture and storage and the impacts on food security impacts [96].

Nevertheless, recoupling responses from trade-off to synergy between SDG 2 and SDG 7 were found over time [93]. Facilities construction of proper clean energy facilities like wind power and solar energy instead of bioenergy normally did not cause extensive damage to farmland [97]. These efforts promote the joint improvement of SDG 7 and SDG 2. There is a chance that the trade-offs between SDG 2 and other SDGs can be transformed into synergies in the future. Subsidizing clean energy to mitigate deforestation by farmers will help protect forests (SDG 15) [93]. A series of emerging trends, such as the growth of the wealthy population (SDG 8), urbanization (SDG 11) and carbon-neutral actions (SDG 13), are likely to further strengthen these connections, linking food security even more closely to other SDGs in China. Sustainable development interventions like healthy nutrition will drastically reduce non-CO_2_ greenhouse gas emissions and then the pressure of land competition caused by climate policies, which are likely to promote trade-off decoupling and synergy coupling between SDG 2 and SDG 13 [98]. Changing the prioritization of management actions can transform trade-offs into synergies, facilitating the overall implementation of the SDGs. Future research should also explore the trade-offs and synergies of food security across transboundary regions, such as the Mekong River Basin, to enhance regional collaboration.

## FUTURE PERSPECTIVES

Based on our synthesis of China’s food security trajectory and systemic challenges, we propose three interconnected research priorities to guide future efforts in enhancing systemic resilience.

First, establishing a predictive, integrated assessment and early-warning system represents a foundational priority. Our analysis reveals that current projections carry uncertainties (±10%–15% for yield forecasts) due to incomplete incorporation of climate extremes, socioeconomic disruptions and adaptation feedback. Strengthening the monitoring and early warning of food security risks is therefore critical. This involves leveraging remote sensing, Internet of Things (IoT) sensors and big data analytics to establish a high spatiotemporal resolution national framework for real-time tracking of crop growth, extreme weather impacts and market volatility. Crucially, this system should integrate seasonal climate outlooks, crop/yield models, data assimilation and machine-learning approaches to deliver short- to seasonal-lead probabilistic forecasts of yields, drought/flood and pest risks, and price dynamics, enabling proactive adaptation. We specifically recommend developing a national food security resilience index that synthesizes the multidimensional drivers identified in this synthesis, enabling real-time monitoring of the balance between domestic production capacity and import dependency under various shock scenarios.

Second, advancing climate-adaptive technologies that also deliver sustainability co-benefits should be prioritized. Our projections indicate divergent crop responses, with wheat yields potentially increasing by 7.0% by the 2050s, while maize yields may decline by 10.1% under the same climate scenarios. This necessitates targeted innovation in climate-resilient agriculture. Rather than promoting specific techniques as universal solutions, research should focus on extracting underlying ecological principles—such as nutrient cycling, biodiversity enhancement and water conservation—to develop adaptable practice packages tailored to different agro-ecological zones [99]. Future research should also quantify the resilience benefits of such adaptive management, particularly their capacity to buffer against the yield variability highlighted in our analysis.

Third, managing food security trade-offs and synergies across boundaries and SDGs represents a cross-cutting priority. Our analysis identifies both strong synergies and notable trade-offs, with these interactions evolving over time. Future research should particularly focus on transboundary dimensions, including virtual water trade, cross-border agricultural investments, and international cooperation mechanisms that can buffer national food systems against domestic production shocks. We specifically recommend developing integrated assessment frameworks capable of quantifying how dietary shifts toward sustainable patterns could simultaneously advance food security, reduce pressure on water resources, and lower agricultural emissions.

Ultimately, strengthening China’s future food security resilience requires an integrated approach that simultaneously addresses resource, environmental and systemic challenges. Achieving this necessitates not only technological innovations but also coordinated policy, institutional reforms and behavioral changes across multiple sectors. China’s experience underscores that enhancing systemic resilience requires a long-term commitment to multidimensional synergistic adaptation. Future strategies should prioritize context-specific solutions that combine advanced technologies with local knowledge, supported by robust monitoring and flexible governance systems. Lessons from China’s journey, particularly its approach to balancing multiple objectives across different resilience stages, can offer valuable guidance for other nations navigating the complex pathway toward sustainable and resilient food systems.

## Supplementary Material

nwaf587_Supplemental_File

## Data Availability

The data that support the findings of this study are available from the corresponding author, Yujie Liu, upon reasonable request.
