# Supplementary material for: Multidimensional synergistic adaptation enhances the systemic resilience in China’s food security
Source: Natl Sci Rev. 2025 Dec 26;13(2):nwaf587. doi: 10.1093/nsr/nwaf587 (PMC12839535; doi:10.1093/nsr/nwaf587)
Supplement: nwaf587_Supplemental_File [file nwaf587_supplemental_file.docx]

**Multidimensional synergistic adaptation enhances the systemic resilience in China’s food security**

**Supplementary Box Food security and its typical indicators**

This review employs a systemic resilience framework to analyse China's food security. We define systemic resilience as the capacity of the food system to anticipate, absorb, recover from, and adapt to shocks and stresses. Multidimensional collaborative adaptation refers to integrated strategies across agricultural, environmental, socioeconomic, and policy domains. Our assessment utilizes both individual and composite indicators, classified into key dimensions as detailed in Supplementary Table 1 and Supplementary Table 2.

Individual indicators focus on specific aspects of food security. On the supply side, per capita grain production and per capita calorie availability are key indicators that reflect grain production and supply and are commonly used in FAO’s food security assessment[4]. Fluctuations in grain production indicate the stability of production and markets and are crucial in the face of extreme weather and climate events, a concern highlighted by the Intergovernmental Panel on Climate Change (IPCC)[5]. The food self-sufficiency rate, which reflects the balance between food production and consumption, is fundamental in maintaining food security and has garnered significant attention from institutions like the World Bank[6-8].

From the demand perspective, per capita disposable income has been a focal point for the World Bank to address food shortages[7]. It indicates the affordability and accessibility of food[9] and is closely related to per capita calorie and protein demand[10]. The Engel coefficient represents the proportion of household income spent on food, with a lower coefficient indicating reduced rates of stunting and thinness[11]. Prevalence of Undernourishment (PoU) reflects insufficient dietary energy intake and the gap between food supply and demand and is widely used by FAO to assess food security[4]. Dietary diversity serves as an indicator of food utilization, reflecting diet quality. An imbalanced diet composition can lead to nutritional deficiencies and impact human health[12, 13]. Additionally, the obesity rate, monitored by the World Health Organization (WHO), signals imbalanced diets and excessive consumption, leading to health problems[14].

However, researchers have reached a consensus that measuring the entire complex structure of food security using a single indicator is challenging. Composite indicators, covering various food security dimensions like the SDG2 score[15] and the Global Food Security Index[16], have gained popularity for assessing food security[17, 18]. For example, the Global Food Security Index, constructed from 68 indicators across dimensions such as affordability, availability, quality and safety, and sustainability and adaptation, provides a comprehensive quantitative and qualitative benchmarking model measuring food security drivers[16].

**Supplementary Table 1. China's food security indicators at the national scale based on four-dimensional FAO indicators.**

| **Dimensions** | **Indicators** | **Source** |
| --- | --- | --- |
| **Availability** | Average dietary energy supply adequacy | FAOSTAT and ESS calculations. |
|  | Share of dietary energy supply derived from cereals, roots and tubers | FAOSTAT and ESS calculations. |
|  | Average protein supply | FAOSTAT |
|  | Average supply of protein of animal origin | FAOSTAT |
|  | Dietary energy supply used in the estimation of prevalence of undernourishment | FAOSTAT and ESS calculations. |
| **Access** | Gross domestic product per capita | World Bank: http://data.worldbank.org/indicator/NY.GDP.PCAP.PP.KD. |
|  | Prevalence of undernourishment | FAOSTAT and ESS calculations. |
|  | Number of people undernourished | FAOSTAT and ESS calculations. |
|  | Rail lines density | World Bank: http://data.worldbank.org/indicator/IS.RRS.TOTL.KM; http://data.worldbank.org/indicator/AG.SRF.TOTL.K2. |
| **Stability** | Cereals imports dependency ratio | FAOSTAT and ESS calculations |
|  | Percent of arable land equipped for irrigation | FAOSTAT and ESS calculations. |
|  | Value of food imports over total merchandise exports | FAOSTAT and ESS calculations. |
|  | Per capita food production variability | FAOSTAT |
|  | Per capita food supply variability | FAOSTAT and ESS calculations. |
| **Utilization** | Prevalence of obesity in the adult population (18 years and older) | World Health Organization Global Health Observatory (GHO) http://apps.who.int/gho/data/node.main.A900A?lang=en |
|  | Prevalence of anemia among women of reproductive age (15-49 years) | World Development Indicators: World Bank: http://data.worldbank.org/indicator/SH.ANM.ALLW.ZS. |
|  | Prevalence of low birthweight | UNICEF and WHO. 2019. Low birthweight estimates, 2019. |

**Supplementary Table 2. The development of of China's food security indicators from Individual to Composite perspective**

| **Indicators type** | | **Indicators** |
| --- | --- | --- |
| Individual indicators | Supply side | Per capita grain production |
|  |  | Per capita calorie availability |
|  |  | Fluctuations in grain production |
|  |  | The food self-sufficiency rate |
|  | Demand side | Per capita disposable income |
|  |  | The Engel coefficient |
|  |  | Prevalence of Undernourishment |
|  |  | Dietary diversity |
|  |  | The obesity rate |
| Composite indicators | SDG2: No Hunger | The score includes nine indicators.: Prevalence of undernourishment, Prevalence of stunting in children under 5 years of age, Prevalence of wasting in children under 5 years of age, Prevalence of obesity, Human Trophic Level, Cereal yield, Sustainable Nitrogen Management Indicator, Yield gap closure, Exports of hazardous pesticides. Data source: https://dashboards.sdgindex.org/ |
|  | Global Food Security Indicator | The Global Food Security Indicator evaluates food security in 113 countries across four key pillars: affordability, availability, quality and safety, and sustainability and adaptation. The indicator is based on a dynamic benchmarking model constructed from 68 qualitative and quantitative drivers of food security. Data source:https://impact.economist.com/sustainability/project/food-security-index/ |

# Supplementary Methods

## Data and Computation

Prevalence of undernutrition and adult obesity was sourced from FAO [19]; all other indicators were from the National Bureau of Statistics of China [20]. Composite food-security scores were compiled from published studies (Supplementary Table 3) and rescaled to a common baseline (2001 = 50). SDG2 scores used in Figs. 1–2 were extracted from published research [15].

## Meta-analysis

We conducted an integrated meta-analysis to improve objectivity and comparability. Searches and inclusion followed a four-step process—identification, screening, eligibility assessment, and inclusion—retaining only China-focused studies with extractable quantitative effects; when results were reported as intervals, the median was used as the period average. Databases covered Web of Science, Google Scholar, CNKI and so on.

The first theme addressed historical drivers and multidimensional adaptation shaping food-security change in China, organized into four classes: agricultural advances (operationalized as the adaptation dimension), climate change (treated as exogenous forcing), socioeconomic change, and land-use change (the latter two may include adaptive responses where framed as management or policy adjustments). We searched from 1980 to 2023 using driver- and outcome-specific keywords (e.g., “agricultural advances,” “climate change,” “socio-economic change,” “land use change,” “yield/production,” and “China”) across major databases, yielding 1,108 records for initial screening. Studies were excluded at title/abstract, study area, or impact-factor stages when they did not meet scope, and qualitative analyses were removed. The final set consisted of quantitative evidence on the impacts of the four driver classes on yields or food-security–related outcomes in China, with effects harmonized by the median-of-range rule where applicable.

The second theme synthesized projected changes in crop yields under future climate change for China’s four major grains—wheat, rice, maize, and soybean. Using climate-keywords paired with crop names and “yield/China,” and setting the window from 2007 (the publication year of the IPCC Fourth Assessment Report) through 31 December 2022, we retrieved 3,008 records. We excluded studies focused solely on extreme events (e.g., floods or droughts), those limited to historical periods, those reporting only national total yields without per-area estimates. We defined a “sample” as the unit-area yield change for a given crop, site or region, scenario, and time slice. The final database contains 828 samples: rice 120, maize 304, wheat 390, and soybean 14. Each entry records the sample identifier, geographic location/site, crop type, scenario, study period, yield change, publication year, and source, with medians used for interval-reported results.

The third theme evaluated linkages between SDG2 (food security) and other SDGs in China. From 176 identified records across the listed databases, we applied standard eligibility rules requiring that the relationship between SDG2 and other SDGs be explicitly clarified. This yielded 44 samples from 21 references (numbers in brackets in our results denote the samples corresponding to each SDG).

**Supplementary Table 3. The published studies and corresponding indicator frameworks for extracting composite food security scores.**

| **Literatures** | **Indicator system** | **Numbers of indicators** | **Year of study** |
| --- | --- | --- | --- |
| Multiscale analysis of factors affecting food security in China, 1980–2017[9] | 1. Per-capita food production 2. Per-capita GDP | 2 | 2000-2017 |
| A Research on the Evaluation of China’s Food Security under the Perspective of Sustainable Development-Based on an Entropy Weight TOPSIS Model[23] | 1. Quantity security 2. Structural security 3. Ecological security of resources 4. Economic security 5. Policy security | 25 | 2001-2020 |
| Research on the evaluation of China’s food security based on entropy weight TOPSIS model (in Chinese)[24] | 1. Supply capacity 2. Supply structure 3. Green development 4. Economic efficiency 5. Basic support | 25 | 2001-2020 |
| The construction, measurement and policy suggestions of Chinese food security evaluation indicator system (in Chinese)[25] | 1. Quantity security 2. Quality security 3. Ecological security 4. Resource security | 8 | 2001-2012 |
| Appraisal and strategic consideration on food security status of China (in Chinese)[26] | 1. Supply 2. Distribution 3. Consumption 4. Efficiency of use 5. Guaranteed result 6. Stability/Vulnerability 7. Sustainability 8. Government regulatory power | 27 | 2000-2011 |
| Systematic appraisal and implementation mechanism research of food security in China (in Chinese)[27] | (1) Quantity security  (2) Ecological security  (3) Nutrition security  (4) Quality security  (5) Price security | 16 | 2000-2014 |
| Recognition and evaluation of food security problems under compound system perspective (in Chinese)[28] | 1. Balance 2. Adaptability 3. Stability 4. Fluidity | 16 | 2000-2014 |
| Analysis of food security and its provincial contribution in mainland China from 1998 to 2016 (in Chinese)[29] | 1. Grain availability 2. Ratio of multi-grain output 3. Stability of grain price 4. Self-sufficiency rate of grain 5. Intensity of arable land input 6. Water resource dependence 7. Energy dependence 8. Intensity of fertilizer use | 8 | 2000-2016 |
| Current situation analysis of food security in China based on fuzzy comprehensive evaluation (in Chinese)[30] | 1. Production safety 2. Consumption safety 3. Circulation safety | 17 | 2001-2017 |

**Supplementary Table 4. China's food supply and demand balance projections for 2019, 2035 and 2050.** Based on China ’s agricultural policy simulation and projection model (CAPSiM), the self-sufficiency rate of major foods (Rice, wheat, maize, soybean, cotton, sugar, pork, beef, mutton, poultry and eggs, milk, aquatic products) in China in 2019, 2035, and 2050. The self-sufficiency rate = (total production + import export) / total demand, and the data is sourced from the model Data from CAPSiM simulations[31, 32].

| Year | Indicators | Rice | Wheat | Maize | Soybean | Cotton | Sugar | Pork | Beef | Mutton | Poultry and eggs | Milk | Aquatic products |
| --- | --- | --- | --- | --- | --- | --- | --- | --- | --- | --- | --- | --- | --- |
| 2019 | Production (10^4^t) | 14673 | 13360 | 26 078 | 1810 | 589 | 1460 | 4255 | 667 | 488 | 3309 | 3201 | 6480 |
|  | Import (10^4^t) | 255 | 349 | 479 | 8851 | 194 | 339 | 313 | 176 | 39 | 0 | 1629 | 624 |
|  | Export (10^4^t) | 275 | 31 | 3 | 12 | 5 | 18 | 21 | 2 | 0 | 10 | 0 | 417 |
|  | Total demand (10^4^t) | 14653 | 13678 | 26554 | 10649 | 778 | 1781 | 4547 | 841 | 527 | 3299 | 4830 | 6687 |
|  | Residential consumption (10^4^t) | 10765 | 9654 | 835 | 10406 | 0 | 1084 | 4547 | 841 | 527 | 3299 | 4830 | 6687 |
|  | Feed grain demand (10^4^t) | 947 | 1363 | 15383 | 70 | 0 | 0 | 0 | 0 | 0 | 0 | 0 | 0 |
|  | Other demand (10^4^t) | 2941 | 2660 | 10337 | 173 | 778 | 697 | 0 | 0 | 0 | 0 | 0 | 0 |
|  | Self-sufficient rate (%) | 100 | 98 | 98 | 17 | 76 | 82 | 94 | 79 | 93 | 100 | 66 | 97 |
| 2035 | Production (10^4^t) | 13137 | 12316 | 27610 | 1904 | 511 | 961 | 6415 | 827 | 576 | 3444 | 3984 | 7878 |
|  | Import (10^4^t) | 271 | 331 | 5609 | 10444 | 270 | 697 | 132 | 489 | 103 | 0 | 2636 | 770 |
|  | Export (10^4^t) | 36 | 33 | 0 | 10 | 4 | 9 | 16 | 1 | 0 | 8 | 0 | 338 |
|  | Total demand (10^4^t) | 13372 | 12614 | 33219 | 12338 | 777 | 1649 | 6531 | 1315 | 679 | 3436 | 6619 | 8310 |
|  | Residential consumption (10^4^t) | 9504 | 9436 | 497 | 12066 | 0 | 959 | 6531 | 1315 | 679 | 3436 | 6619 | 8310 |
|  | Feed grain demand (10^4^t) | 607 | 1228 | 19572 | 69 | 0 | 0 | 0 | 0 | 0 | 0 | 0 | 0 |
|  | Other demand (10^4^t) | 3261 | 2950 | 13151 | 203 | 777 | 959 | 0 | 0 | 0 | 0 | 0 | 0 |
|  | Self-sufficient rate (%) | 98 | 98 | 83 | 15 | 66 | 58 | 98 | 63 | 85 | 100 | 60 | 95 |
| 2050 | Production (10^4^t) | 11413 | 10976 | 30490 | 1881 | 422 | 568 | 6933 | 918 | 618 | 3238 | 4172 | 8716 |
|  | Import (10^4^t) | 220 | 283 | 6624 | 10470 | 365 | 1005 | 167 | 988 | 202 | 0 | 3959 | 827 |
|  | Export (10^4^t) | 44 | 39 | 0 | 10 | 3 | 6 | 13 | 0 | 0 | 7 | 0 | 314 |
|  | Total demand (10^4^t) | 11589 | 11220 | 37114 | 12340 | 784 | 1567 | 7087 | 1905 | 821 | 3231 | 8132 | 9229 |
|  | Residential consumption (10^4^t) | 7609 | 6834 | 261 | 12034 | 0 | 856 | 7087 | 1905 | 821 | 3231 | 8132 | 9229 |
|  | Feed grain demand (10^4^t) | 355 | 1056 | 22558 | 64 | 0 | 0 | 0 | 0 | 0 | 0 | 0 | 0 |
|  | Other demand (10^4^t) | 3625 | 3331 | 14295 | 243 | 784 | 7111 | 0 | 0 | 0 | 0 | 0 | 0 |
|  | Self-sufficient rate (%) | 98 | 98 | 82 | 15 | 84 | 36 | 98 | 48 | 75 | 100 | 51 | 94 |

Note: (1) Based on 70% rice yield; (2) Based on 12% sugar yield.

# Supplementary Figures

.
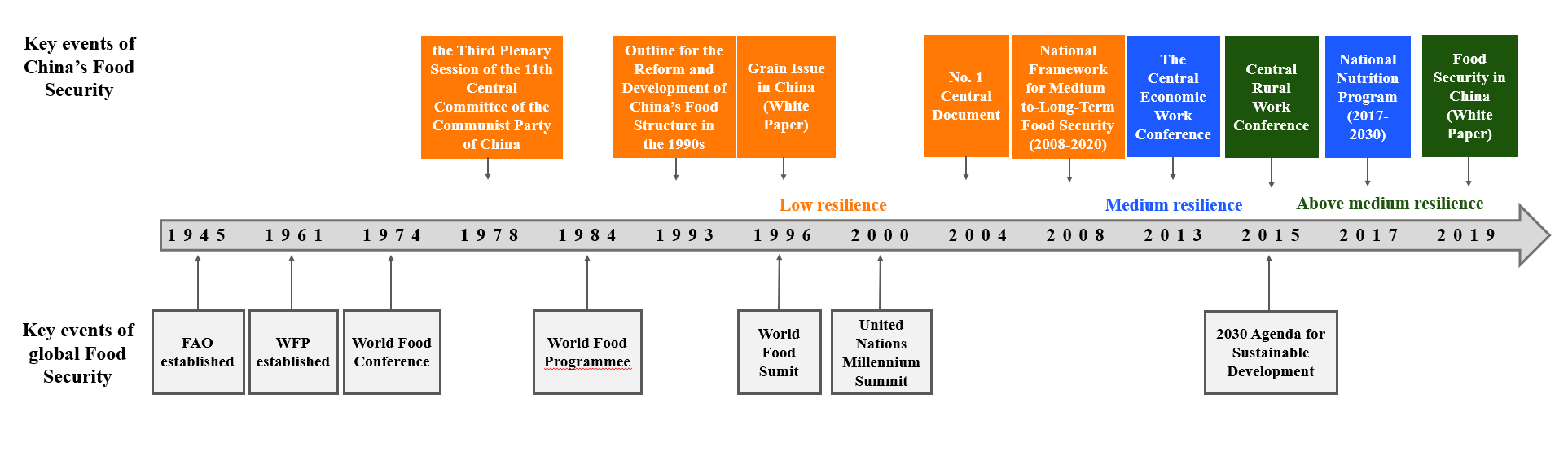


**Supplementary Figure 1｜**Different stages of food security development and key indicators of concern. Based on the key events in the development process of the world and China’s food security, the development of Chinese food security is divided into three different stages with different focuses: eating enough, eating well and eating sustainably. The key indicators receiving attention at different stages are different. It should be noted that in the ‘eating sustainably’ stage, the issue of ‘eating well’ is still a key concern for China's food security.


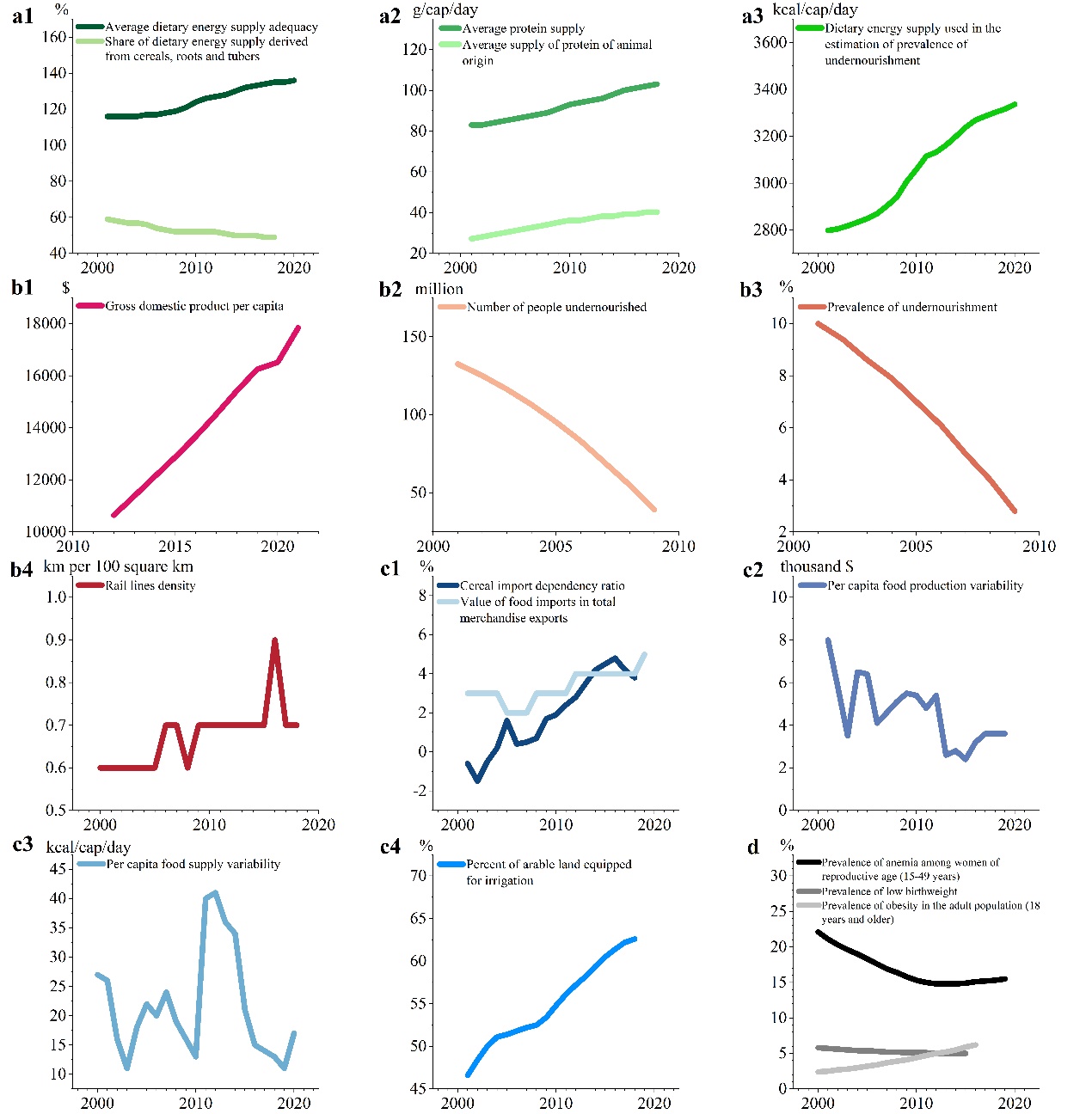


Supplementary Figure 2｜**Changes in China's food security at the national scale based on four-dimensional FAO indicators.** The selection of indicators is based on the data of China in the FAO database, and refers to the four-dimensional indicator system, including availability (figure **a1-3**), access (figure **b1-4**), stability (figure **c1-4**), and utilization (figure **d**).

**
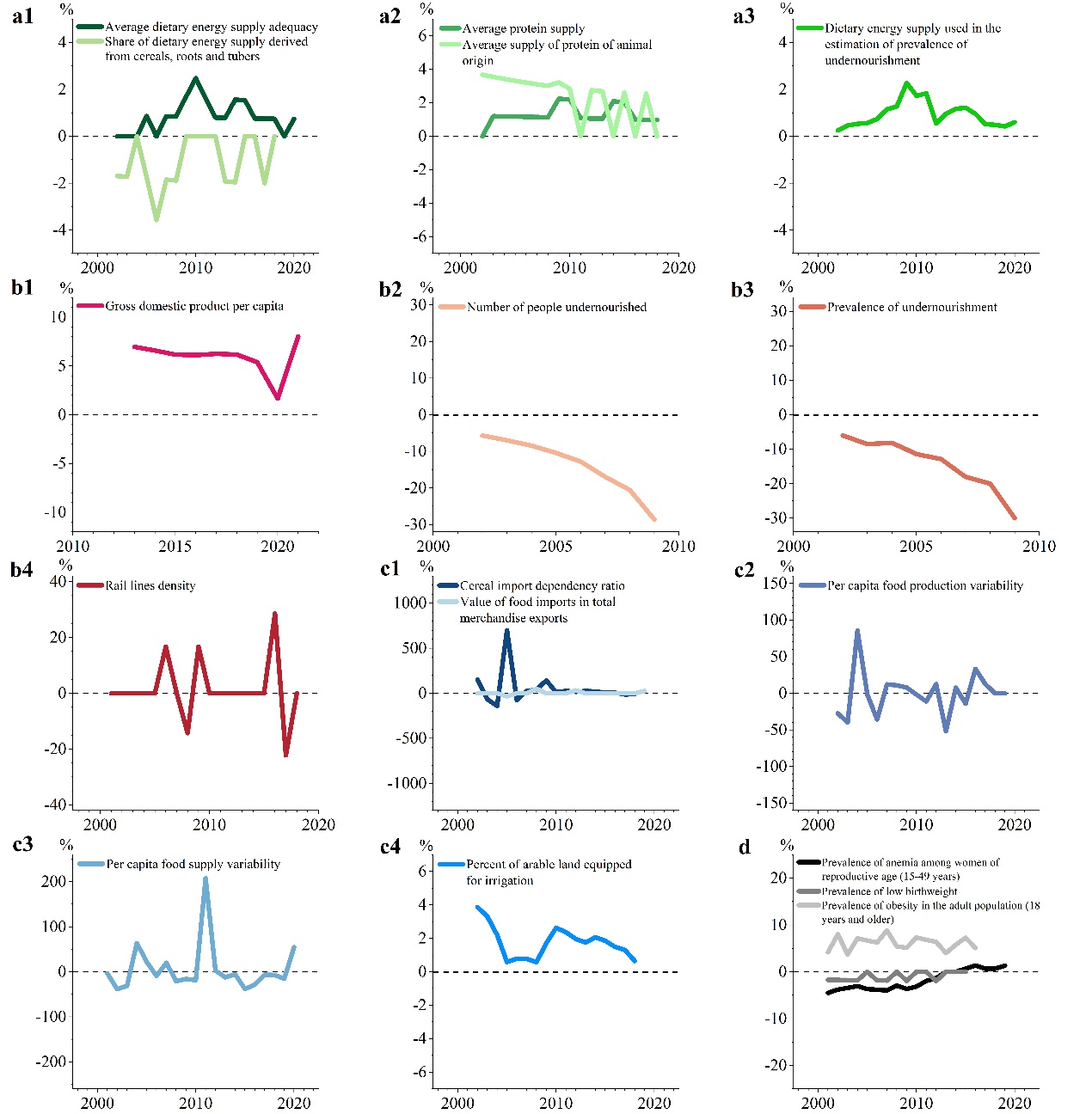
**

Supplementary Figure 3｜**The year-on-year growth rate of indicators at the national scale based on FAO’s four-dimensional system.** The selection of indicators is the same as Supplementary Fig. 2, including availability (figure **a1-3**), access (figure **b1-4**), stability (figure **c1-4**), and utilization dimensions (figure **d**).


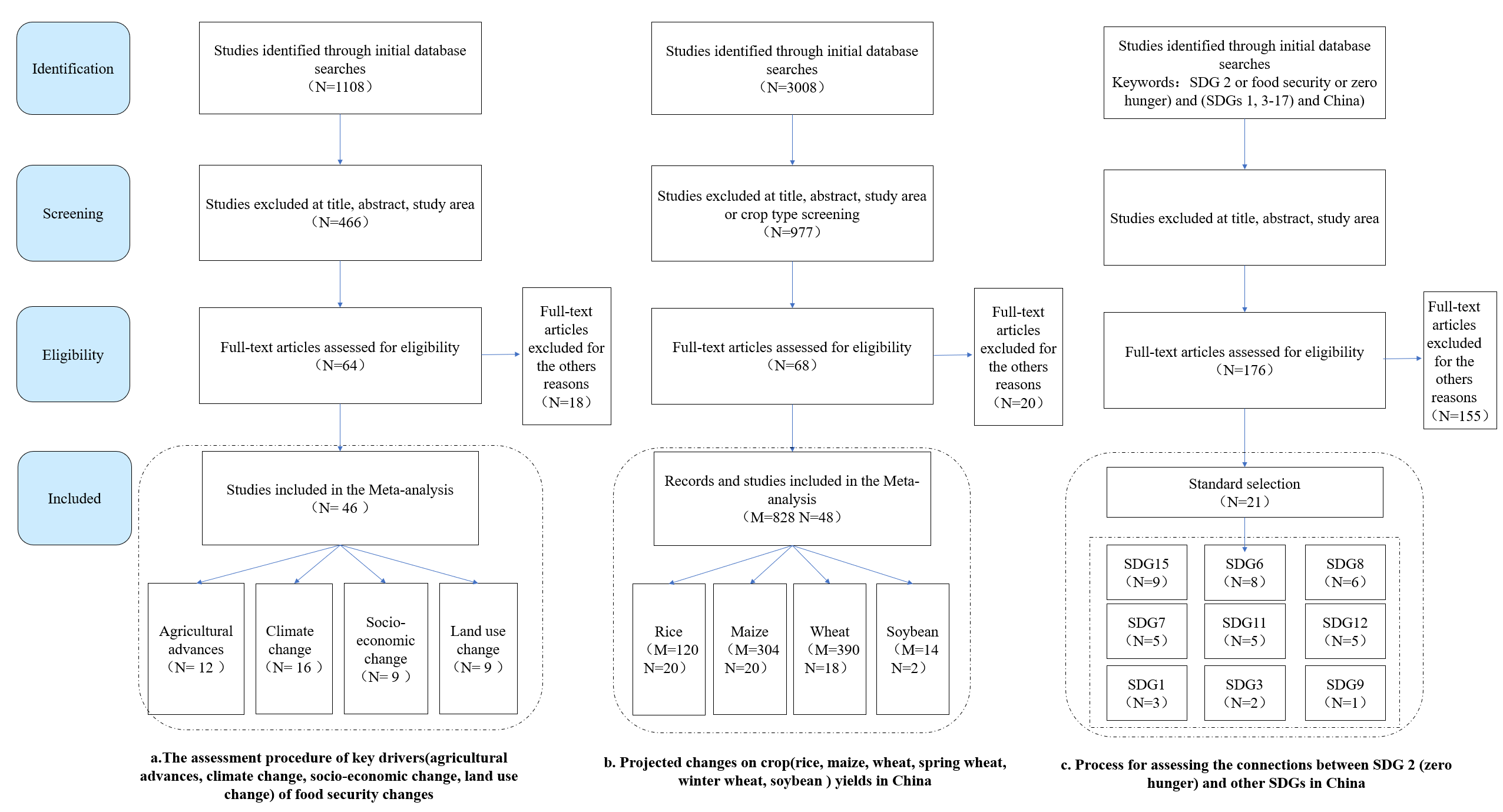


Supplementary Figure 4｜**Study selection flow diagram (Preferred Reporting Items for Meta-Analysis).** a｜The assessment procedure of key drivers(agricultural advances, Climate change, Socio-economic change, land use change) of food security changes. b｜Projected changes on crop(rice, maize, wheat, spring wheat, winter wheat, soybean ) yields in China. c｜Process for assessing the connections between SDG 2 (food security) and other SDGs in China.

# References

1. FAO I, UNICEF, WFP and WHO. *The State of Food Security and Nutrition in the World 2023*. Rome: FAO; 2023. (Urbanization, agrifood systems transformation and healthy diets across the rural–urban continuum) (DHHS publication no.: Report Number)| (GPO o. Document Number)|.

2. Upton JB, Cissé JD, Barrett CB. Food security as resilience: reconciling definition and measurement. *Agricultural economics*. 2016; **47**(S1): 135-147.

3. HLPE. *Food security and nutrition: building a global narrative towards 2030*. Rome: 2020. (A report by the High Level Panel of Experts on Food Security and Nutrition of the Committee on World Food Security) (DHHS publication no.: Report Number)| (GPO o. Document Number)|.

4. Barrett CB. Measuring Food Insecurity. *Science*. 2010; **327**(5967): 825-828. doi: 10.1126/science.1182768

5. IPCC. *Climate Change 2022: Impacts, Adaptation and Vulnerability*. 2022 (DHHS publication no.: Report Number)| (GPO o. Document Number)|.

6. Niu Y, Xie G, Xiao Y *et al.* Spatiotemporal patterns and determinants of grain self-sufficiency in China. *Foods*. 2021; **10**(4): 747. doi: 10.3390/foods10040747

7. Ross-Larsen. *At China's table : food security options*2010.

8. Deng X, Yue T, Liu Y *et al.* Changes in China's food self-sufficiency rate in the context of a changing dietary structure. *J Glob Inf Manag*. 2021; **30**(6): 1-19.

9. Lv F, Deng L, Zhang Z *et al.* Multiscale analysis of factors affecting food security in China, 1980-2017. *Environmental Science and Pollution Research*. 2022; **29**(5): 6511-6525. doi: 10.1007/s11356-021-16125-1

10. Tilman D, Balzer C, Hill J *et al.* Global food demand and the sustainable intensification of agriculture. *P Natl Acad Sci USA*. 2011; **108**(50): 20260-20264. doi: 10.1073/pnas.1116437108

11. Dong Y, Jan C, Ma Y *et al.* Economic development and the nutritional status of Chinese school-aged children and adolescents from 1995 to 2014: an analysis of five successive national surveys. *Lancet Diabetes & Endocrinology*. 2019; **7**(4): 288-299. doi: 10.1016/s2213-8587(19)30075-0

12. Kearney J. Food consumption trends and drivers. *Philosophical Transactions of the Royal Society B-Biological Sciences*. 2010; **365**(1554): 2793-2807. doi: 10.1098/rstb.2010.0149

13. Willett W, Rockstrom J, Loken B *et al.* Food in the Anthropocene: the EAT-Lancet Commission on healthy diets from sustainable food systems. *Lancet*. 2019; **393**(10170): 447-492. doi: 10.1016/s0140-6736(18)31788-4

14. Drewnowski A, Popkin BM. The nutrition transition: New trends in the global diet. *Nutrition Reviews*. 1997; **55**(2): 31-43.

15. Xu Z, Chau SN, Chen X *et al.* Assessing progress towards sustainable development over space and time. *Nature*. 2020; **577**(7788): 74-78. doi: 10.1038/s41586-019-1846-3

16. EIU. Global Food Security Index. The Economist Intelligence Unit <https://impact.economist.com/sustainability/project/food-security-index#introduction>. 2021.

17. Coates J. Build it back better: Deconstructing food security for improved measurement and action. *Global Food Security*. 2013; **2**(3): 188-194. doi: <https://doi.org/10.1016/j.gfs.2013.05.002>

18. Bongaarts J. The State of Food Security and Nutrition in the World 2020. Transforming food systems for affordable healthy diets. *Population and Development Review*. 2021; **47**(2): 558-558.

19. Huang Y, Tian X. Food accessibility, diversity of agricultural production and dietary pattern in rural China. *Food Policy*. 2019; **84**: 92-102.

20. Wang Y. The challenges and strategies of food security under rapid urbanization in China. *Sustainability*. 2019; **11**(2): 542.

21. Challinor AJ, Watson J, Lobell DB *et al.* A meta-analysis of crop yield under climate change and adaptation. *Nature climate change*. 2014; **4**(4): 287-291.

22. Ruehr S, Keenan TF, Williams C *et al.* Evidence and attribution of the enhanced land carbon sink. *Nat Rev Earth Environ*. 2023; **4**(8): 518-534.

23. Zhang XY, Wang Y, Bao J *et al.* A research on the evaluation of China's food security under the perspective of sustainable development-based on an entropy weight TOPSIS model. *Agriculture-Basel*. 2022; **12**(11): 1926. doi: 10.3390/agriculture12111926

24. Zhang XY, Bao J, Xu SW. Research on the evaluation of China’s food security based on entropy weight TOPSIS model (in Chinese). *Chinese Journal of Agricultural Resources and Regional Planning*. 2023; **1**: 1-11.

25. Yang JL, Lei YK. The construction, measurement and policy suggestions of Chinese food security evaluation index system (in Chinese). *Rural Economy*. 2014; **5**(05): 23-27.

26. Zhang YH, Liu CQ, Guo LL. Appraisal and strategic consideration on food security status of China (in Chinese). *China Rural Survey*. 2015; **1**(01): 2-14.

27. P. L. Systematic appraisal and implementation mechanism research of food security in China (in Chinese). Chinese Academy of Agricultural Sciences, PhD dissertation (2016).

28. Tang S, Zhang JC, Li LF. Recognition and evaluation of food security problems under compound system perspective (in Chinese). *Statistics & Decision*. 2016; **7**(07): 42-46. doi: 10.13546/j.cnki.tjyjc.2016.07.010

29. Jiang JC, Zhao LS, Zhen DF *et al.* Analysis of food security and its provincial contribution in mainland China from 1998 to 2016 (in Chinese). *Journal of Liaoning Normal University (Natural Science Edition)* 2018; **41**(03): 395-402.

30. Sun CW, Zhang J, Gu BT *et al.* Current situation analysis of food security in China based on fuzzy comprehensive evaluation (in Chinese). *Shanxi Agricultural Economy*. 2019; **15**(15): 2-4. doi: 10.16675/j.cnki.cn14-1065/f.2019.15.001

31. Xie W, Huang J, Wang J *et al.* Climate change impacts on China's agriculture: The responses from market and trade. *China Economic Review*. 2020; **62**: 101256.

32. Niu Y, Xie G, Xiao Y *et al.* The story of grain self‐sufficiency: China's food security and food for thought. *Food and Energy Security*. 2022; **11**(1): e344.
